# A siphonous morphology affects light-harvesting modulation in the intertidal green macroalga *Bryopsis corticulans* (Ulvophyceae)

**DOI:** 10.1007/s00425-018-2854-5

**Published:** 2018-02-19

**Authors:** Vasco Giovagnetti, Guangye Han, Maxwell A. Ware, Petra Ungerer, Xiaochun Qin, Wen-Da Wang, Tingyun Kuang, Jian-Ren Shen, Alexander V. Ruban

**Affiliations:** 1 School of Biological and Chemical Sciences, Queen Mary University of London, Mile End Road, London, E1 4NS UK; 2 Photosynthesis Research Center, Key Laboratory of Photobiology, Institute of Botany, Chinese Academy of Sciences, Beijing, 100093 China; 3 Research Institute for Interdisciplinary Science, Graduate School of Natural Science and Technology, Okayama University, 3-1-1 Tsushima, Naka, Okayama, 700-8530 Japan

**Keywords:** *Bryopsis corticulans*, Intertidal algae, Light harvesting, LHCSR, Non-photochemical quenching, PsbS

## Abstract

The macroalga *Bryopsis corticulans* relies on a sustained protective NPQ and a peculiar body architecture to efficiently adapt to the extreme light changes of intertidal shores.

During low tides, intertidal algae experience prolonged high light stress. Efficient dissipation of excess light energy, measured as non-photochemical quenching (NPQ) of chlorophyll fluorescence, is therefore required to avoid photodamage. Light-harvesting regulation was studied in the intertidal macroalga *Bryopsis corticulans*, during high light and air exposure. Photosynthetic capacity and NPQ kinetics were assessed in different filament layers of the algal tufts and in intact chloroplasts to unravel the nature of NPQ in this siphonous green alga. We found that the morphology and pigment composition of the *B. corticulans* body provides functional segregation between surface sunlit filaments (protective state) and those that are underneath and undergo severe light attenuation (light-harvesting state). In the surface filaments, very high and sustained NPQ gradually formed. NPQ induction was triggered by the formation of transthylakoid proton gradient and independent of the xanthophyll cycle. PsbS and LHCSR proteins seem not to be active in the NPQ mechanism activated by this alga. Our results show that *B. corticulans* endures excess light energy pressure through a sustained protective NPQ, not related to photodamage, as revealed by the unusually quick restoration of photosystem II (PSII) function in the dark. This might suggest either the occurrence of transient PSII photoinactivation or a fast rate of PSII repair cycle.

## Introduction

Oxygenic photosynthesis uses sunlight energy to convert carbon dioxide and water into carbohydrates and molecular oxygen, through the transfer of electrons between photosystems II and I (PSII and PSI) (Blankenship 2014). This process generates an electrochemical proton gradient across the thylakoid membranes that is used for the synthesis of adenosine triphosphate (ATP) and reduced nicotinamide adenine dinucleotide phosphate (NADPH), and feedback modulation of light harvesting (Cardol et al. 2011).

Photosynthetic light energy conversion takes place in PSII and I, multi-subunit pigment–protein complexes that are embedded in the thylakoid membrane. These complexes are composed of a core complex, containing the reaction centre (RC) and inner antenna complexes, and a light-harvesting complex (LHC) or peripheral antenna, built of many pigment–protein complexes (Ruban et al. 2012; Croce and van Amerongen 2014). While RCs are responsible for charge separation and electron transfer, light-harvesting antennae are specialised to absorb light in a wide spectral range and increase the effective absorption cross section of RCs. Despite the fact that RCs of PSII and PSI differ from each other (Amunts et al. 2007; Umena et al. 2011; Qin et al. 2015a; Suga et al. 2015), the individual photosystems and their core antennae are highly conserved in all oxygen-evolving organisms (Blankenship et al. 2007; Hohmann-Marriott and Blankenship 2011). In contrast, peripheral light-harvesting systems are modular units diversified among photosynthetic groups of organisms. These systems display marked differences in pigment–protein composition/organisation and size to match the diversity in light intensity and quality of different habitats (Falkowski and Raven 2007; Ruban et al. 2011; Ballottari et al. 2012; Blankenship 2014).

Sudden and extreme changes in light intensity and quality that occur in nature affect photosynthetic rates. When light energy is absorbed in excess, it can oversaturate the electron transport chain capacity and associated enzyme-catalysed reactions without driving photochemistry. This increases the chance of photo-oxidative damage (Aro et al. 1993; Ruban et al. 2012). Under high light, the concurrent presence of excess excitation energy and O_2_ within the photosynthetic membranes can yield reactive oxygen species (e.g. singlet oxygen) due to the transfer of energy from chlorophyll (Chl) triplet states (^3^Chl*) to O_2_, ultimately causing photoinhibition (Aro et al. 1993; Tyystjärvi and Aro 1996; Giovagnetti and Ruban 2015). Photosynthetic organisms have developed sophisticated mechanisms to cope with fluctuating light environments and reduce the accumulation of detrimental excess light energy, thus balancing the input and utilisation of sunlight in photosynthesis (Li et al. 2009; Goss and Lepetit 2015; Ruban 2016).

The fastest and reversible process activated to protect against high light can be measured at room temperature as a decline in PSII antenna Chl fluorescence yield and is referred to as non-photochemical quenching of Chl fluorescence (NPQ) (Ruban 2016). Although NPQ is essential and common to all photosynthetic organisms, clear differences in the underlying mechanisms have been observed (see Demmig-Adams et al. 2014; Goss and Lepetit 2015; Ruban 2016). Overall, NPQ reflects a number of mechanistically different processes, namely: energy-dependent (qE), state-transition (qT), photoinhibitory (qI) and zeaxanthin-dependent (qZ) quenching components (Krause and Weis 1991; Ruban et al. 2012; Demmig-Adams et al. 2014; Goss and Lepetit 2015; Ruban 2016). While kinetic criteria are usually employed to discern between them (i.e. times of formation and relaxation), it has been recently underlined how this approach can give misleading interpretations if we are to investigate their effective protective nature (Ruban and Murchie 2012; Giovagnetti and Ruban 2015).

qE is the largest and fastest response to light intensity changes (forming and recovering within minutes) and it is often the most effective in protecting PSII RCs against photodamage (Ruban and Murchie 2012; Demmig-Adams et al. 2014; Goss and Lepetit 2015; Ruban 2016). It depends on the generation of a transthylakoid proton gradient (ΔpH, Krause and Behrend 1986; Noctor et al. 1993), and in vascular plants it is controlled by the activation of the protein PSII subunit S (PsbS) through the acidification of the thylakoid lumen (Li et al. 2000, 2004) and the operation of the (violaxanthin–antheraxanthin–zeaxanthin) xanthophyll cycle (XC, Yamamoto and Kamite 1972; Yamamoto 1979). Both PsbS and zeaxanthin act as allosteric modulators that enhance the sensitivity of PSII antenna to lumenal protons and regulate antenna conformational changes (Ruban et al. 2012; Sacharz et al. 2017). The presence of specific polypeptides and the XC involvement in NPQ vary among the eukaryotic photosynthetic lineages so far studied. In the green alga *Chlamydomonas reinhardtii*, the stress-related members of the LHC protein superfamily, LHCSR3 and LHCSR1, were proposed to play the roles of pH sensor and active site of quenching (Peers et al. 2009; Bonente et al. 2011; Ballottari et al. 2016; Dinc et al. 2016), while PsbS was quickly and transiently accumulated (Tibiletti et al. 2016). Whereas both PsbS and LHCSR are active during NPQ in the moss *Physcomitrella patens* (Alboresi et al. 2010), some of the LHCX isoforms, related to the LHCSR protein family, are involved in the modulation of excess energy dissipation in the diatom *Phaeodactylum tricornutum* (Bailleul et al. 2010; Lepetit et al. 2013, 2016; Taddei et al. 2016). Whilst the role of de-epoxidation of violaxanthin into zeaxanthin is still debated in *C. reinhardtii* (Bonente et al. 2011; Dinc et al. 2016), NPQ has often been reported to be XC activation dependent in mosses (Pinnola et al. 2013) and diatoms (the latter contain the diadinoxanthin–diatoxanthin XC as the main XC; see Goss and Lepetit 2015).

In this study, we investigated the regulation of light harvesting in the marine green alga *Bryopsis corticulans* (class Ulvophyceae, order Bryopsidales) (Graham and Wilcox 2000; Lam and Zechman 2006; Verbruggen et al. 2009). *B. corticulans* algae typically flourish in rocky intertidal shores during summer, where they are adapted to extreme light fluctuations and variations in other abiotic environmental factors (e.g. temperature, inorganic carbon and nutrient availability) due to the seasonal tidal rhythm (Davison and Pearson 1996; Gómez and Huovinen 2011; Hurd et al. 2014). This species is able to survive repeated and prolonged periods either underwater (~ 1–2 m) at low/moderate light or exposed to the atmosphere at very high light. This must therefore require fine-tuning of the light-harvesting capacity during the day to adapt to light limitation when submerged and prevent photo-induced damage when exposed to the atmosphere (Li et al. 2009; Ruban et al. 2012; Ruban 2016). Moreover, *B. corticulans* response to high light stress occurs without the involvement of XC pigments active in NPQ (Wang et al. 2013), as reported for the algal order of Bryopsidales (Christa et al. 2017).

In line with the typically described body structure of Bryopsidales, *B. corticulans* is a macroscopic, siphonous green alga that comprises a single, giant cell with tubular filaments called ‘siphons’. Its algal body (thallus) consists of a tough, flexible cell wall that surrounds a continuous cytoplasm containing multiple nuclei, discoid plastids, and other organelles (Graham and Wilcox 2000; Lam and Zechman 2006). The photosynthetic apparatus of *B. corticulans* exhibits peculiar properties when compared with that of higher plants. Although substantial differences in the composition of Chls and carotenoids have been reported between LHCII isolated from *B. corticulans* and *Spinacia oleracea* (Liu et al. 2004; Wang et al. 2013), their polypeptide sequences and key residues responsible for maintaining LHCII structure and function were highly conserved (Wang et al. 2013). Lutein and violaxanthin are not present in LHCII of siphonous algae; however, carotenoids with conjugated carbonyl groups, e.g. siphonaxanthin (Sx) and siphonein (S), are found together with more Chl *b* molecules, enhancing the absorption of blue-green and green light (Nakayama and Okada 1990; Chen et al. 2005, 2008; Wang et al. 2013). This is particularly advantageous when thriving in intertidal waters, where visible light is strongly attenuated, apart from the blue-green light region (Kirk 2011).

Additionally, the isolation and characterisation of a PSI–LHCI supercomplex and its sub-complexes revealed a number of unique features. Notably, *B. corticulans* possesses a unique α- and ε-carotene-type PSI core complex among photosynthetic eukaryotes, suggesting for the first time a structural flexibility of the PSI core (Qin et al. 2015b). Moreover, it was found that seven different carotenoids bind to the Lhca antennae, conferring a high carotenoid/Chl ratio, and less red Chls are present in the PSI–LHCI supercomplex relative to that of *S. oleracea* (Qin et al. 2015a, b).

Given the (1) unique pigment (and potentially protein) composition of PSII and PSI light-harvesting systems and (2) the flexible adaptation to the intertidal light environment, here we studied the light-harvesting and photoprotection modulation in *B. corticulans* algae during low tides and exposure to the atmosphere. The results obtained are discussed in the light of the current understanding of the NPQ molecular mechanisms revealed in other eukaryotic photosynthetic O_2_ evolvers.

## Materials and methods

### Study site and collection of algae

**Fig. 1:**
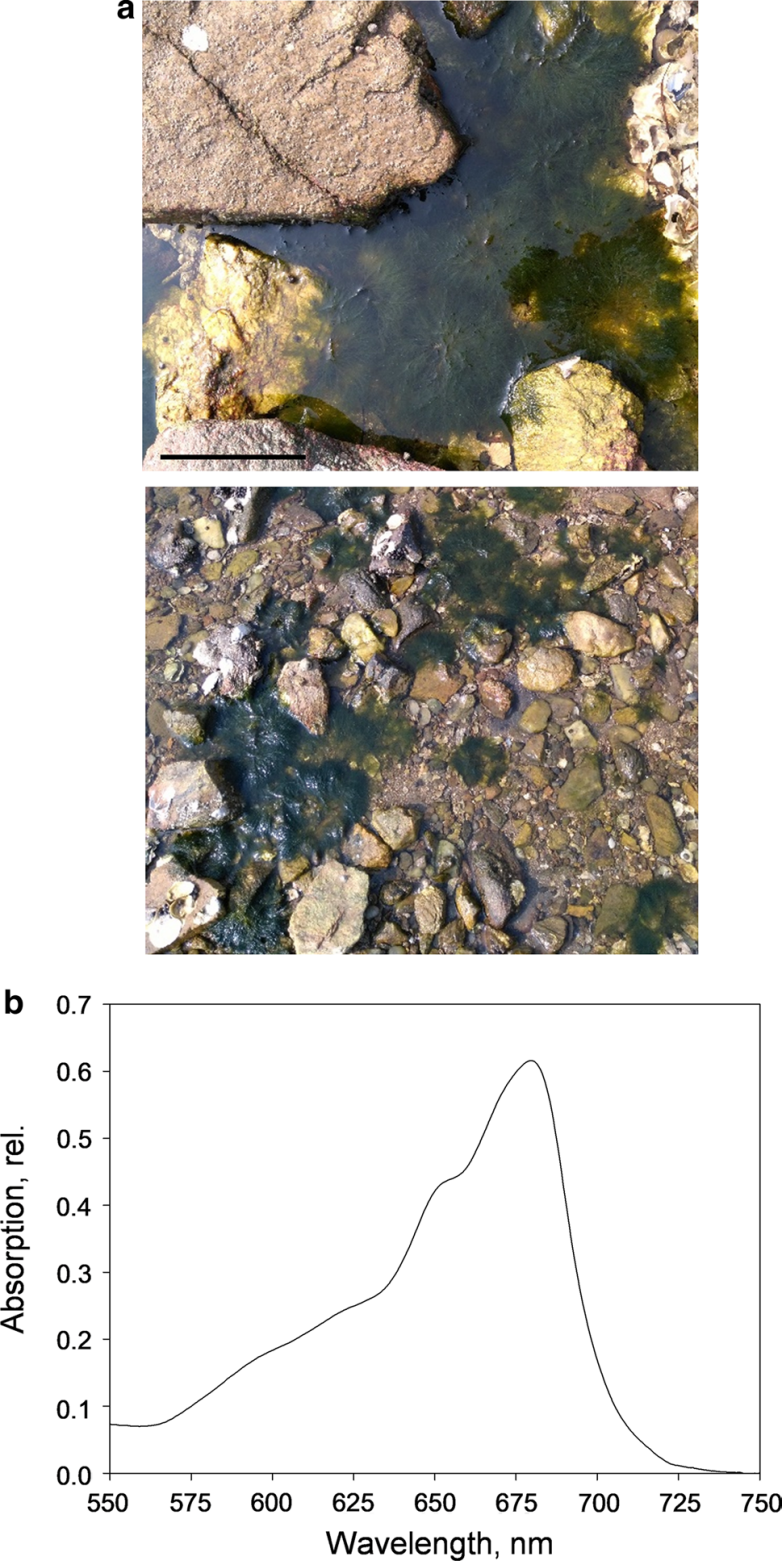
*B. corticulans* algae during emersions due to low tides (**a**) and absorption capacity (**b**). Absorption spectra of single layers of adjacent filaments were measured at room temperature, after 30 min dark adaptation. The spectrum presented is the average of four repeats. Scale bar in **a**, 10 cm

*Bryopsis corticulans* Setchell (Ulvophyceae) (Collins et al. 1899) algae populating the rocky intertidal shores of Yantai (north-eastern province of Shandong, China; 37°28′35″N, 121°26′27″E) were collected during summer (September 2016). Field work was carried out during low tides, causing algae to be partly or completely exposed to air (Fig. 1a). Algae were collected during the lowest tide level and measurements were taken between 11:00 and 15:00 hours, after which the water level gradually increased and algal tufts became submerged underwater (~ 1–2 m below the water surface). While algae submersions lasted until early morning (low or moderate light levels), the prolonged exposure to the atmosphere coincided with the highest light intensities and temperatures of the day. Experiments were carried out in the field and in the laboratory, which was in close proximity to the sampling site. For the laboratory experiments, freshly collected samples were maintained for short time periods in filtered natural seawater. After dark adaptation, samples were used to perform Chl fluorescence measurements and chloroplast isolation from *B. corticulans*.

### Chloroplast preparation and pigment analysis

Intact chloroplasts were isolated after 30 min dark adaptation. All the following steps were carried out in dim light and at 4 °C. Five fresh algal tufts were ground in 100 mL of pre-cooled homogenisation buffer (10 mM Mes pH 6.5, 2 mM KCl, 5 mM EDTA, 1 M sorbitol). Algae were ground by three consecutive cycles (3 s each) using a household blender. The homogenate was filtered through four layers of muslin, followed by four layers of muslin and one layer of cotton wool. The filtrate was centrifuged for 8 min at 4000*g* (4 °C). The supernatant was removed and the chloroplast pellet washed in a resuspension buffer (10 mM Mes pH 6.5, 2 mM KCl, 5 mM EDTA, 0.33 M sorbitol). Chloroplasts showed a high degree of intactness, as verified under the microscope, and *F*_v_/*F*_m_ values of ~ 0.73, i.e. slightly lower than values measured in *B. corticulans* filaments (see the text). Total Chl content and Chl *a*/*b* ratio were determined in 80% acetone according to Porra et al. (1989). High-performance liquid chromatography (HPLC) was performed as described in Wang et al. (2013).

### Absorption spectroscopy

Absorption spectra were recorded with a Shimadzu UV–Visible 2550 spectrophotometer (Kyoto, Japan) at room temperature. Measurements were performed on single layers of adjacent filaments of (30 min) dark-adapted algae, which were carefully placed on a glass slide under the microscope. The diameter of each single filament was 0.26 ± 0.02 mm. Empty spaces between filaments were carefully avoided to provide a homogenous surface for the spectrophotometer beam.

### Chlorophyll fluorescence analysis

Chl fluorescence was measured on *B. corticulans* filaments using a Walz JUNIOR-PAM fluorometer (Walz Effeltrich, Germany), equipped with a magnetic leaf clip. For the field experiments, algal tufts of *B. corticulans* were dark adapted for 30 min prior to their exposure to actinic light (AL). Rapid light curves were assessed using ten gradually increasing intensities of AL, each lasting 10 s and followed by a saturating pulse (SP). 24 repeats were taken, with error bars representing ± SEM. Relative photosynthetic efficiency (*α*), electron transport rate (ETR) and light-saturation parameter (Ek) were calculated by the Software WinControl (Walz) fitting the model of Platt et al. (1980).

To assess the relevance of *B. corticulans* morphology to light-harvesting regulation, laboratory experiments were carried out. Chl fluorescence quenching induction and relaxation were measured on dark-adapted whole algal tufts, each one submerged in filtered seawater contained in a beaker (see Fig. 3a for a diagram). Given the static floating of the algae, a stratification of the algal body filaments was obtained along the water volume. Chl fluorescence was probed in both ‘surface filaments’ (exposed to the atmosphere) and ‘bottom filaments’ (2 cm underneath the water surface; Fig. 3a). White AL (2000 µmol photons m^−2^ s^−1^) was provided by two external light sources, whereas measuring light (ML) and SP (0.6 s and 4000 µmol photons m^−2^ s^−1^) were provided by two JUNIOR-PAM fibre optics, which were secured to prevent movement (Fig. 3a). The entire procedure consisted of 1 h of AL illumination (NPQ induction), followed by 3 h of recovery in the dark (NPQ relaxation). An SP was applied every 5 min during light treatment, and 10 s after AL was switched off, as well as every 20 min in the dark. For both layers, four independent measurements were performed.

Chl fluorescence was measured on *B. corticulans* intact chloroplasts with a DUAL-PAM-100 fluorometer (Walz Effeltrich, Germany), equipped with the liquid cell adapter. Measurements were carried out on 2 mL of intact chloroplasts (final concentration of 35 µM of total Chl in the resuspension buffer) in a quartz cuvette. Constant, gentle stirring was applied. To induce quenching, AL intensities of 211 µmol photons m^−2^ s^−1^ (5 min), or 760 and 2500 µmol photons m^−2^ s^−1^ (30 min) were used. Where mentioned, 100 µM nigericin or 600 µM diaminodurene (DAD) was added in the dark (2 min before ML was switched on) to intact chloroplasts to prevent or increase the formation of ΔpH across the thylakoid membrane, respectively (Wraight and Crofts 1970; Johnson and Ruban 2011). The use of relatively high concentration of both uncouplers was needed for effective penetration through the chloroplast surrounding membrane (Grant and Wright 1980). AL and SP were provided by an array of 635 nm light-emitting diodes. *F*_o_ and *F*_o_′ (i.e. the fluorescence level with PSII RCs open in the dark and during AL exposure) were measured in the presence of a 7 µmol photons m^−2^ s^−1^ measuring beam. *F*_s_ is the steady state fluorescence level under AL. The maximum fluorescence in the dark-adapted state (*F*_m_), during the course of AL illumination (*F*_m_′), and in the subsequent dark relaxation periods (*F*_m_′′) was determined using 0.6 s SP of 4000 µmol photons m^−2^ s^−1^. Maximum quantum yield of PSII in the dark was calculated as *F*_v_/*F*_m_ = [(*F*_m_–*F*_o_)/*F*_m_], photochemical quenching as *q*_P_ = (*F*_m_′–*F*_s_)/(*F*_m_′–*F*_o_′) and non-photochemical fluorescence quenching as NPQ = [(*F*_m_/*F*_m_′) − 1]. The photochemical quenching measured in the dark immediately after illumination (*q*_Pd_) was calculated as *q*_Pd_ = (*F*_m_′ − *F*_o_′_act._)/(*F*_m_′ − *F*_o_′_calc._) (Ruban and Murchie 2012). *F*_o_′_act._ and *F*_o_′_calc._ are the measured and calculated levels of minimum fluorescence in the dark after illumination, respectively. *F*_o_′_calc._ is calculated according to Oxborough and Baker (1997) as *F*_o_′_act.=_1/(1/*F*_o_–1/*F*_m_ + 1/*F*_m_′). Under high light, *F*_o_′_act._ becomes greater than *F*_o_′_calc._, and *q*_Pd_ declines below 1.00. This reflects photodamage of PSII RCs, which has been considered to occur when more than 2% of PSII RCs are closed (*q*_Pd_ < 0.98) (Ruban and Murchie 2012; Giovagnetti and Ruban 2015).

### SDS-PAGE and western blot analysis

To analyse potential changes in polypeptide composition upon NPQ formation/relaxation, glycine–sodium dodecyl sulphate polyacrylamide gel electrophoresis (glycine–SDS-PAGE; Laemmli 1970) was used, as in Qin et al. (2015b). Stacking and separation gels of 4 and 12% acrylamide/bis-acrylamide (29:1) mix, respectively, were employed. Samples were denatured with a lithium dodecyl sulphate sample buffer (Ikeuchi and Inoue 1988) at 40 °C for 10 min, and 10 μg Chl of chloroplasts was loaded per lane. After electrophoresis, gels were used for electroblotting onto nitrocellulose membrane (GE Healthcare, UK) and the proteins were incubated overnight at 4 °C with antibodies raised against PsbS (Agrisera AS09533, 1:2000), LHCSR3 (Agrisera AS142766, 1:1000) and LHCSR1 (Agrisera AS142819, 1:1000). The β subunit of ATP synthase (ATP-B, Agrisera AS05085, 1:2000) was used as loading control. Enhanced chemiluminescence (WSE-6200HLuminiGraph II chemiluminescent imaging system, ATTO, Japan) was used to visualise protein bands after incubation with a horseradish peroxidase (HRP)-conjugated secondary antibody (Agrisera AS09602, 1:20000).

### Statistical analysis

Statistical significance at a *P* value < 0.05 was determined with Student’s *t* test, using a two-tailed distribution. Error bars represent the standard error of the mean (SEM = ${\text{SD}}/\sqrt n$). The results of the statistical analysis are given in the text and figure legends.

## Results

### Photosynthetic capacity of *B. corticulans* algae during low tides

Because of tidal cycles, intertidal algae experience continuous switching between air exposure and water submersion (Fig. 1a; Madsen and Maberly 1990; Davison and Pearson 1996). Exposure to the atmosphere poses problems of water loss and desiccation, nutrient availability, and very high light absorption, all factors that can ultimately impair photosynthesis and cause photodamage. We therefore investigated the photosynthetic properties and capacity of *B. corticulans* algal tufts in the field, when they partially or completely emerged from water during low tides (Fig. 1a). Measurements took place when the air light intensity and temperature reached the highest levels of the day, demanding optimisation of light harvesting (up to ~ 2000 µmol photons m^−2^ s^−1^ and ~ 33 °C at midday).

A 30 min dark adaptation was found to be sufficient to relax the excess light excitation pressure encountered in nature by the *B. corticulans* algae. Indeed, a PSII maximum quantum yield in the dark (*F*_v_/*F*_m_) of 0.76 ± 0.01 was measured. This is in agreement with values previously reported for other green macroalgae belonging to the order of Ulvales (Franklin et al. 1992; Henley et al. 1991; Henley 1992; Yamazaki et al. 2005) and Bryopsidales (Yamazaki et al. 2005; Cruz et al. 2014), as well as for several different species of Bryopsidales cultured in the laboratory under constant low light (i.e. 25 µmol photons m^−2^ s^−1^ with a 12 h light/12 h dark photoperiod; Christa et al. 2017). The intertidal *B. corticulans* was not specifically investigated in the work of Christa et al. (2017), where they found lower *F*_v_/*F*_m_ values (~ 0.62) when some of those species were grown under higher light intensity (i.e. 200 µmol photons m^−2^ s^−1^), due to absence of a full recovery of sustained NPQ components.

*Bryopsis corticulans* siphonous morphology consists of branched thalli that form free-floating, intensely green tufts of filaments (Graham and Wilcox 2000). Such filaments are interconnected and thickly amassed, especially when fully exposed to the atmosphere (Fig. 1a). The absorption spectra measured on single thin layers of filaments (with filament diameter of 0.26 ± 0.02 mm) showed a high, maximal optical density (OD) of 0.62 ± 0.03 in the Q_y_ region of Chl *a* and a significant contribution of Chl *b* (at ~ 650 nm; Fig. 1b). It should be noted that OD ~ 0.6 corresponds to a reduction in incident light intensity of ~ 75% (Ruban 2012). The high Chl *b* content is in line with previous spectroscopic and biochemical studies on LHCII from *B. corticulans* (Chen et al. 2005, 2008; Wang et al. 2013) and the particularly low Chl *a*/*b* ratio (1.26 ± 0.03) of chloroplasts isolated in this study. These results indicate the presence of a large light-harvesting antenna efficiently adapted to shaded conditions (Anderson and Osmond 1987; Anderson et al. 1988), rather than the high light experienced during emersions.

**Fig. 2:**
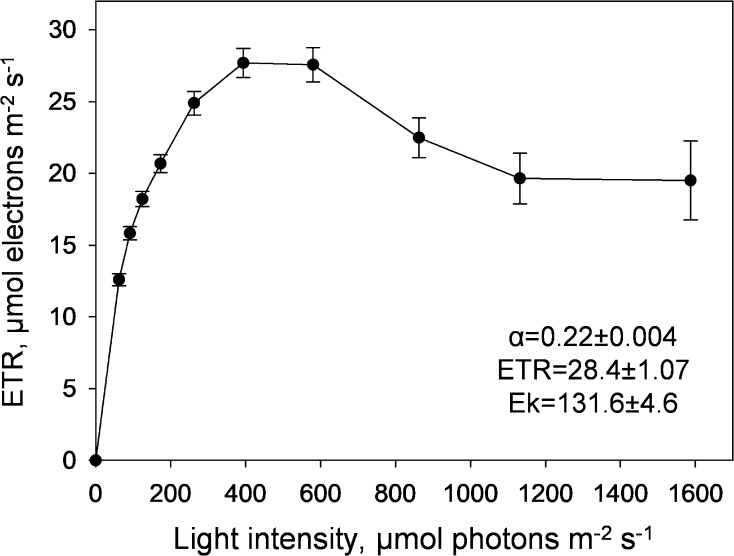
Rapid light curves measured on freshly collected *B. corticulans* algal tufts. Algae were dark adapted for 30 min, after which ten gradually increasing intensities of actinic light were applied, each lasting 10 s and followed by a saturating pulse. Data are averages of 24 repeats ± SEM. Relative photosynthetic efficiency (*α*), electron transport rate (ETR) and light-saturation parameter (Ek) were calculated by fitting the model of Platt et al. (1980)

The relative electron transport rates (ETR), assessed on dark-adapted algal tufts in the field through rapid light curves (RLC; Platt et al. 1980), support the shade adaptation of this macroalga (Fig. 2). With a substantially lower saturation light of photosynthesis (Ek ~ 130 µmol photons m^−2^ s^−1^) than the high light intensities endured during emersion, maximum ETR was 28.4 ± 1.07 µmol electrons m^−2^ s^−1^ and saturated at ~ 400 µmol photons m^−2^ s^−1^, after which loss of PSII function was observed (Giovagnetti and Ruban 2015) (Fig. 2). RLC-retrieved parameters here presented are in agreement with other reports on intertidal green macroalgae during low tides (e.g. Gómez et al. 2004; Wang et al. 2012; Holzinger et al. 2015).

### Roles played by the algal body morphology of *B. corticulans* in photoprotection

Given the morphology and absorption properties of the filament clusters of *B. corticulans*, we investigated if the ‘algal body architecture’ could itself function in the photoprotective strategy adopted against excess light exposure. Laboratory experiments were thus designed to investigate how the canopy effect produced by the siphonous morphology modulates NPQ induction and relaxation, and photochemical quenching (q_P_).

**Fig. 3:**
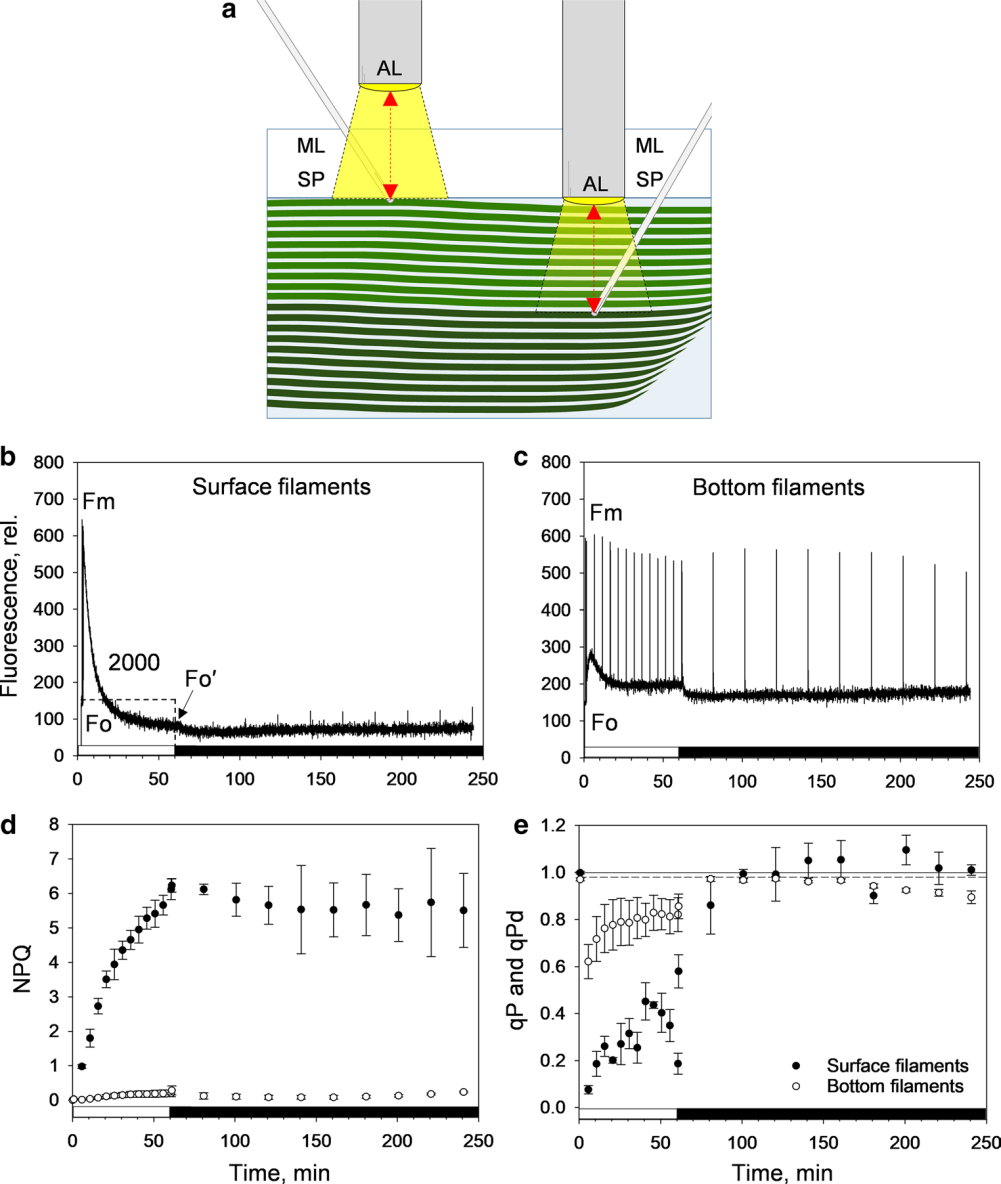
Diagram of the experimental setup used in the laboratory (**a**) to assess chlorophyll fluorescence induction and relaxation in surface and bottom filaments of *B. corticulans* (**b**, **c**). Representative traces are shown for surface (air exposed, **b**) and bottom filaments (2 cm underneath the surface layer and underwater, **c**). Evolution of non-photochemical fluorescence quenching (NPQ, **d**) and photochemical fluorescence quenching over time (*q*_P_ and *q*_Pd_, **e**; see Ruban and Murchie 2012). Closed and open circles represent surface and bottom filaments, respectively (**d**, **e**). Filaments were illuminated by two external light probes, with white actinic light (AL) intensity of 2000 µmol photons m^−2^ s^−1^. Measuring light (ML) and saturating light pulses (SP, 0.6 s and 4000 µmol photons m^−2^ s^−1^) were provided by two JUNIOR-PAM fibre optics, which were secured to prevent movement. 1 h of AL illumination (white bars) was followed by 3 h of recovery in the dark (black bars). An SP was applied every 5 min during light treatment, and 10 s after the actinic light was switched off, as well as every 20 min in the dark. For both layers, four independent measurements were performed. Averages are four repeats ± SEM. *F*_*m*_ maximum fluorescence in the dark, and *F*_*o*_ *and F*_*o*_′ minimum fluorescence in the dark and after AL exposure, respectively

Freshly collected, whole algal tufts were dark adapted and placed in a small beaker, submerged in filtered seawater. Chl fluorescence quenching was measured either on ‘surface filaments’ exposed to the air, or ‘bottom filaments’ that were 2 cm underwater below the algal surface layer (see Fig. 3a and refer to “Materials and methods” for the experimental setup). While the surface filaments of the algal thallus were illuminated by high actinic light intensity (2000 µmol photons m^−2^ s^−1^, for 1 h), the same light intensity on the bottom filaments was strongly attenuated as a result of the absorption of the overlying filaments floating in the water (Figs. 1b, 3a). The experimental setup prevented accurate measurements of the light attenuation. However, it should be noted that if evenly absorbing filaments were closely attached, the light intensity would have exponentially decreased to zero in less than ten filaments (given an OD ~ 0.6 per single filament of ~ 0.25 mm diameter; Fig. 1b).

As predicted, we found a strong quenching at the surface layer of filaments (Fig. 3b), but almost none in the filaments underneath (Fig. 3c). NPQ induction was barely visible in the bottom filaments (Fig. 3d), and while the occurrence of state transitions cannot be ruled out (Fig. 3c), qT was not investigated in the present work. In contrast, at the surface, NPQ gradually formed over 1 h of illumination, reaching very high levels (6.1 ± 0.3, Fig. 3d). Moreover, *F*_o_ quenching occurred, indicating the presence of an antenna-associated NPQ (Horton and Ruban 1993) in *B. corticulans*, as it is currently, widely accepted for higher plants and other photosynthetic organisms (Ruban et al. 2012; Goss and Lepetit 2015). However, NPQ was sustained, almost without any recovery during prolonged darkness (Fig. 3d), and its formation was not related to XC activity, since zeaxanthin was not detected in chloroplasts (Table 1; different light intensities were used to modulate the extent of NPQ). Only a minor and stable amount of violaxanthin was found together with several major carotenoids, including the typical Sx and S (Table 1) (Chen et al. 2008; Wang et al. 2013; Qin et al. 2015b). Both results agree with a recent study revealing the absence of the fast and reversible qE component in Bryopsidales, where NPQ was found to be ΔpH independent and not related to XC (Christa et al. 2017).

**Table 1:** Carotenoid content of *B. corticulans* chloroplasts

Light conditions	Sx/Car	S/Car	Sx/S	Vx/Car	NPQ
Dark	0.209 ± 0.001	0.174 ± 0.001	1.200 ± 0.002	0.054 ± 0.0003	0.0 ± 0.0
Low light	0.212 ± 0.001	0.175 ± 0.001	1.212 ± 0.003	0.054 ± 0.0002	0.2 ± 0.02
Moderate light	0.224 ± 0.001	0.179 ± 0.001	1.250 ± 0.005	0.053 ± 0.001	1.01 ± 0.01
High light	0.240 ± 0.009	0.190 ± 0.008	1.264 ± 0.005	0.055 ± 0.001	2.01 ± 0.01

Chloroplasts were either dark adapted or exposed to low (211 μmol photons m^−2^ s^−1^, 5 min), moderate (760 μmol photons m^−2^ s^−1^, 30 min) and high light intensity (2500 μmol photons m^−2^ s^−1^, 30 min) to induce non-photochemical quenching (NPQ). The corresponding NPQ values are shown in the table. Carotenoid changes are presented as the ratio between relative integrated peak areas of HPLC chromatograms. Data are mean ± SEM of three repeats

*Car* total carotenoid content (siphonaxanthin, all-*trans* neoxanthin, 9′-*cis* neoxanthin, violaxanthin, siphonein, ε-carotene and α-carotene), *S* siphonein, *Sx* siphonaxanthin, *Vx* violaxanthin

**Fig. 4:**
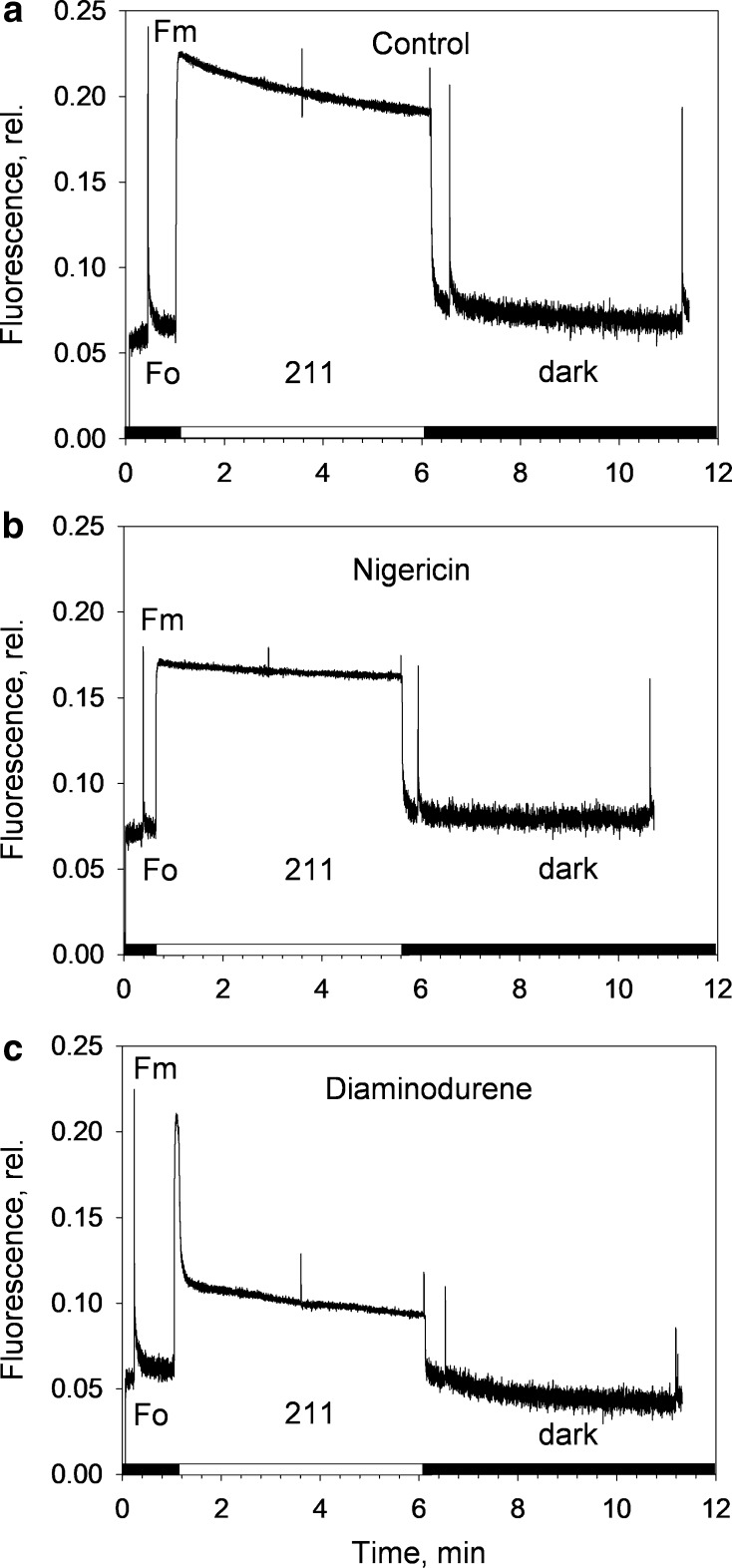
Representative traces of chlorophyll fluorescence induction and relaxation from *B. corticulans* chloroplasts. Measurements were carried out on 2 ml of intact chloroplasts (final concentration of 35 µM of Chl in resuspension buffer) in a quartz cuvette. Constant, gentle stirring was applied. Actinic light intensity was 211 µmol photons m^−2^ s^−1^. 100 µM nigericin or 600 µM diaminodurene was added in the dark to prevent or enhance the formation of ΔpH across the thylakoid membrane, respectively. Illumination treatment lasted for 5 min, followed by 5 min in the dark. An SP was applied after 2 and 5 min during light treatment, and after 10 s and 5 min in the dark. Three to four independent measurements were performed. *F*_*m*_ maximum fluorescence in the dark, *F*_*o*_ minimum fluorescence in the dark

In relation to the clear diversity in NPQ kinetics observed, *q*_P_ significantly differed between surface and bottom filaments during illumination (*P* < 0.05, Fig. 3e). In the surface filaments, a rapid drop in *q*_P_ was initially seen as the actinic light was switched on, followed by a gradual increase during most of the remaining illumination period. This suggests that NPQ formation released the excitation pressure in the antenna, until the last 10 min of illumination, when *q*_P_ decreased again (*q*_P_ ~ 0.2, Fig. 3e). Surprisingly, while the first average *q*_Pd_ values (taken after 10 s of darkness) were significantly different between the two conditions (*q*_Pd_ ~ 0.6 and ~ 0.86 in surface and bottom filaments, respectively; *P* < 0.05), no significant difference was found in *q*_Pd_ between the two layers during the first 80 min of recovery in the dark (*P* > 0.05, Fig. 3e). Moreover, *q*_Pd_ recovered to 1.00 in ~ 40 min (Fig. 4b), which is a much faster kinetics than that observed in *A. thaliana* leaves (Ruban and Belgio 2014). Although the NPQ process could not fully prevent photodamage in filaments exposed to high light, the loss of PSII function was quickly recovered in the dark, regardless of the lack of NPQ fast reversibility (Fig. 3d). This underlines the protective effectiveness of the antenna-based, sustained NPQ that was activated in filaments exposed to high light.

From 140 min (i.e. after 80 min in the dark), *q*_Pd_ values became again significantly different between the two conditions (*P* < 0.05), potentially because of the occurrence of some antenna detachment in the surface filaments, as indicated by the slight rise in *q*_Pd_ above 1.00 (Fig. 3e; see Giovagnetti and Ruban 2015; Ware et al. 2015). However, we are not able to explain the decrease in *q*_Pd_ below 0.98 measured in the bottom filaments, which is unrelated to photodamage, since samples were already in the dark for 2 h and PSII functionality was not impaired after the light treatment (Fig. 3e). A possible rearrangement of antenna pigment–protein complexes in these shaded filaments might have affected *q*_Pd_ during prolonged darkness.

Overall, we reveal the key role played by the siphonous morphology in light-harvesting regulation and adaptation to the intertidal light environment in *B. corticulans*, based on the presence of a slowly reversible, protective NPQ, which does not relate to photodamage.

### Functional insights into the NPQ mechanism of *B. corticulans*

The sustained NPQ in other species belonging to the same order as *B. corticulans* has been proposed not to be triggered by the thylakoid lumen acidification (Christa et al. 2017). To see if this also holds for *B. corticulans*, chloroplasts were isolated and the Chl fluorescence kinetics in nigericin- and DAD-treated samples were monitored and compared to control untreated ones (Fig. 4). The uncoupler nigericin was used to collapse the ΔpH across thylakoid membrane, whereas DAD increased the levels of ΔpH by stimulating cyclic electron flow around PSI. Both molecules are widely used and known to alter qE (Wraight and Crofts 1970; Johnson and Ruban 2011).

**Fig. 5:**
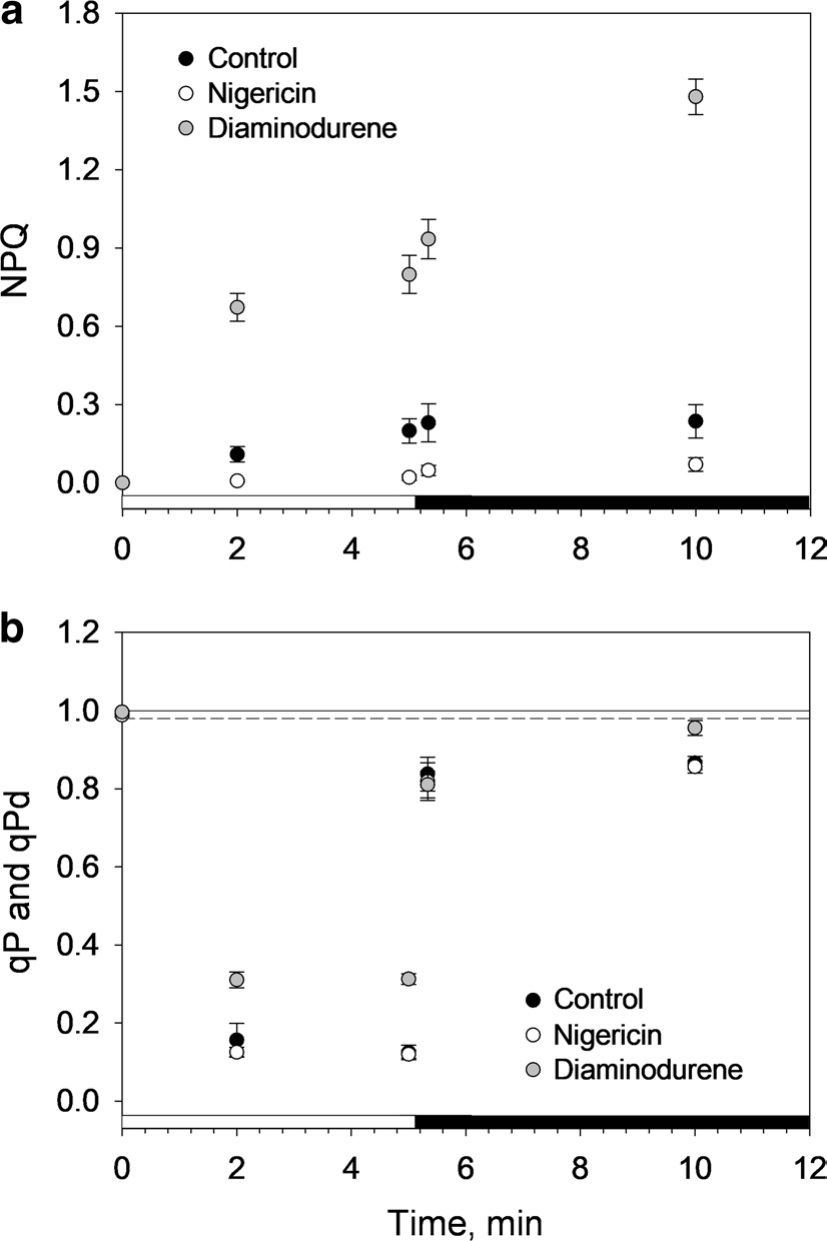
Evolution of non-photochemical fluorescence quenching (NPQ, **a**) and photochemical fluorescence quenching over time (*q*_P_ and *q*_Pd_, **b**) on *B. corticulans* chloroplasts. Control (black circles), nigericin- (white circles) and diaminodurene (DAD)-treated samples (grey circles) were illuminated with an actinic light intensity of 211 μmol photons m^−2^ s^−1^ for 5 min. Recovery in the dark lasted 5 min. Averages are three to four repeats ± SEM

**Fig. 6:**
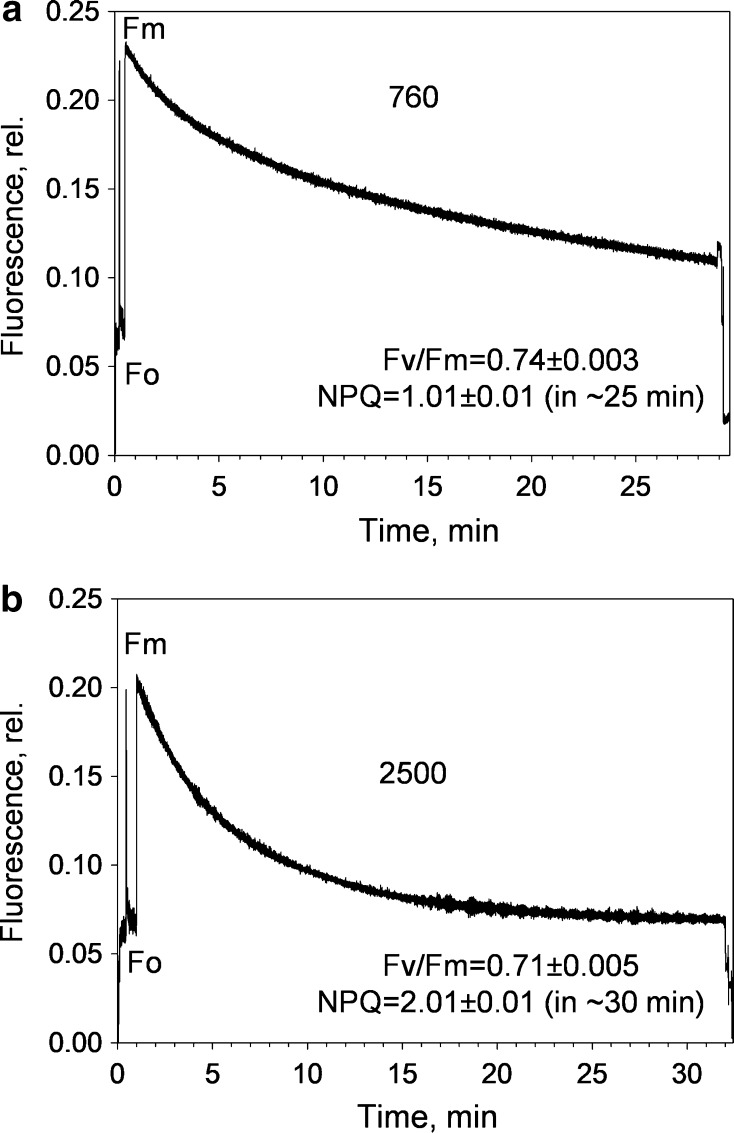
Representative traces of chlorophyll fluorescence induction from *B. corticulans* chloroplasts. Measurements were carried out on 2 mL of intact chloroplasts (final concentration of 35 µM of Chl in resuspension buffer) in a quartz cuvette. Constant, gentle stirring was applied. High actinic light intensities (760 and 2500 µmol photons m^−2^ s^−1^, 30 min) were used to induce high NPQ. Three to four independent measurements were performed, and the corresponding *F*_v_/*F*_m_ and NPQ data are averages of the three to four repeats ± SEM

Figure 4 depicts representative fluorescence traces from intact chloroplasts (*F*_v_/*F*_m_ ~ 0.73) in response to 5 min illumination followed by 5 min in the dark. Disruption (nigericin, Fig. 4b) or enhancement (DAD, Fig. 4c) of the transmembrane proton gradient controlled the extent of quenching accordingly. After light treatment, NPQ was ~ 4 times higher when chloroplasts were treated with DAD, in comparison to the control, while nigericin prevented NPQ formation (Fig. 5a). The low NPQ levels measured were related to the low actinic light and short treatment applied (i.e. 211 µmol photons m^−2^ s^−1^ for 5 min). Larger NPQ values were indeed achieved under greater light intensities and longer treatment (~ 30 min, Fig. 6; Table 1). Regardless of the low actinic light, NPQ was not able to relax during darkness in control and DAD-treated samples (Fig. 5a), but it rather increased, in agreement with the sustained nature of the NPQ revealed in vivo in this study (Fig. 3b, d) and in Bryopsidales (and Christa et al. 2017). When nigericin was added, NPQ was just measurable, but exhibited a similar, slight increase during dark relaxation as in control and DAD conditions (Fig. 5a). Thus, NPQ is triggered by the initial lumen acidification in *B. corticulans* chloroplasts, which possibly initiates a reorganisation of the antenna (Ruban et al. 2012) that does not reverse quickly in the dark. This fits with the alga adaptation to prolonged emersions. Moreover, the larger the NPQ, the lower is the excitation pressure of the antenna, resulting in the fastest and highest *q*_Pd_ recovery in DAD-treated chloroplasts after 5 min of dark recovery (0.96 ± 0.02, Fig. 5b). *q*_Pd_ recovery was instead comparable between control and nigericin treatment (Fig. 5b). The fact that enhancing NPQ clearly reduced photodamage of chloroplasts further proves the protective nature of the sustained NPQ formed by *B. corticulans*.

**Fig. 7:**
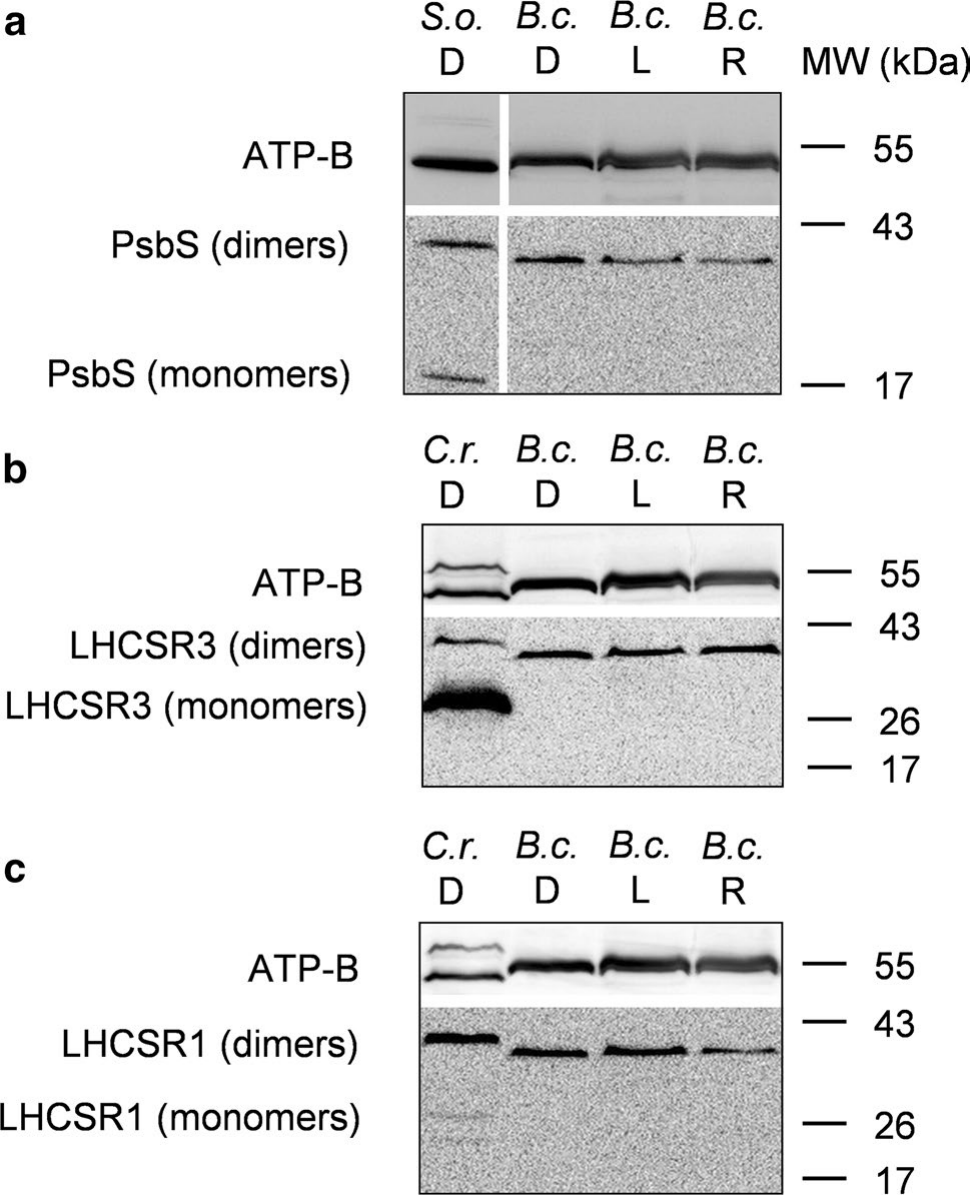
Western blot analysis of PsbS and LHCSR proteins from dark-adapted (D, 30 min), light-treated (L, 2500 µmol photons m^−2^ s^−1^, 30 min) and post-dark-recovery chloroplasts (R, 1 h) isolated from *B. corticulans*. Samples were analysed by immunoblotting with an anti-PsbS (**a**), anti-LHCSR3 (**b**) and anti-LHCSR1 antibodies (**c**). Control samples are dark-adapted *S. oleracea* (**a**) and *C. reinhardtii* chloroplasts (**b**, **c**). Note that *B. corticulans* and *S. oleracea* samples have been probed against anti-PsbS antibody in the same membrane. The separation between lanes is only due to the presence of different samples between those presented of *S. oleracea* and *B. corticulans* (**a**). The *β* subunit of ATP synthase (ATP-B) was used as loading control. 10 µg of Chl was loaded in each lane

Specific proteins control qE in higher plants (PsbS) and other green algae (e.g. PsbS and LHCSR in *C. reinhardtii*) (Bonente et al. 2011; Ruban 2016; Tibiletti et al. 2016). However, the presence of PsbS and LHCSR is yet to be confirmed in Bryopsidales (Handrich et al. 2017), possibly in line with the reported lack of a flexible qE (Christa et al. 2017). Since biochemical studies are clearly needed to exclude their occurrence (Handrich et al. 2017), we performed western blotting employing anti-PsbS and anti-LHCSR3/1 antibodies. *B. corticulans* chloroplasts were either dark adapted, illuminated (NPQ = 2.3 ± 0.25) or dark adapted for 1 h after illumination (recovery NPQ = 1.9 ± 0.23; Fig. 7). Dark-adapted chloroplasts of *S*. *oleracea* and *C. reinhardtii* were used as controls (Fig. 7).

Monomers of PsbS (~ 20 kDa) were only found in *S*. *oleracea* (PsbS, Fig. 7a), whereas monomers of LHCSR3 and LHCSR1 (~ 26 kDa) were found in *C. reinhardtii* (Fig. 7b, c). The presence of both LHCSR isoforms in *C. reinhardtii* samples was achieved by growing the algae under high light. Bands suggesting the presence of PsbS dimers (~ 40 kDa) and LHCSR3/LHCSR1 oligomers (MW ~ 42 kDa, potentially LHCSR dimers) were also visible in *S*. *oleracea* and *C. reinhardtii*, respectively (Fig. 7). Most relevantly, no monomer of either of the proteins was detected in *B. corticulans* (Fig. 7), but only their dimeric or oligomeric states, regardless of light/dark treatment (Fig. 7). This was not altered when denaturing samples at room temperature, instead of heating them at 40 °C for 10 min. Bands corresponding to PsbS dimers have been already shown in higher plants despite the use of denaturing SDS-PAGE (Bergantino et al. 2003; Sacharz et al. 2017), while LHCSR dimer formation has been seen in *C. reinhardtii* by native electrophoresis (Bonente et al. 2011). At the same time, caution should be taken when using antibodies whose reactivity has been only confirmed in other model species. For this reason, unspecific binding of antibodies cannot be fully excluded at this stage.

## Discussion

Life in the intertidal ecosystems demands a precise regulation of photosynthesis to adapt to the rapidly changing light environment. For intertidal algae, the timing of atmosphere or water exposure interacts with diel extreme changes in light and other essential abiotic factors (Madsen and Maberly 1990; Davison and Pearson 1996; Hurd et al. 2014). Nonetheless, high diversity and density of algae are often found in intertidal rocky habitats, implying they evolved a flexible, robust photosynthetic machinery capable of withstanding such abiotic challenges.

We studied the siphonous green macroalga, *B. corticulans*, which belongs to the cosmopolitan order of Bryopsidales, ecologically relevant for being major primary producers in tropical marine habitats and notorious invasive species (e.g. *Codium fragile* and *Caulerpa taxifolia*) (Graham and Wilcox 2000; Provan et al. 2004; Verbruggen et al. 2009). In particular, we address the regulation of light harvesting and photoprotection when *B. corticulans* tufts, formed by multiple filaments, experience high light stress during low tides.

Our data show that *B. corticulans* possesses a large light-harvesting antenna and is a shade-adapted species (Anderson and Osmond 1987; Anderson et al. 1988), in agreement with observations made in other marine green macroalgae (Anderson et al. 1980; Anderson 1983; Yamazaki et al. 2005). The Chl *a*/*b* ratios here measured in chloroplasts (~ 1.26) are substantially lower than those of thylakoid membranes of shade-adapted (2.0–2.2) and light-adapted plants (2.6–3.6) (Anderson et al. 1988; Ware et al. 2015). The presence of such a large antenna is also consistent with the measured *F*_v_/*F*_m_ values, lower than those reported in plants. When submerged in turbid, intertidal waters, *B. corticulans* algae grow and fix inorganic carbon under low light, where the reflection at the water surface and absorption/scattering by particles dissolved or suspended in the water affect the light quality available (Kirk 2011). In these conditions, not only the size, but also the pigment composition of *B. corticulans* antenna complexes provides an ecological advantage. Being equipped with the class-specific carotenoids, Sx and S (Nakayama and Okada 1990; Chen et al. 2005, 2008; Wang et al. 2013), *B. corticulans* can indeed exploit the spectral region of blue-green light (Kirk 2011).

Given this enhanced capacity to absorb a limiting amount of photons underwater, high light must be in turn safely dissipated during complete or partial exposure to air, as light becomes oversaturating. In these conditions, *B. corticulans* is able to efficiently cope with excess light energy absorption by adopting a ‘locked’ sustained protective state of the light-harvesting systems in filaments that are directly subjected to excess light. A very high NPQ is measured in these surface filaments, which forms gradually and is independent of XC operation (here and Wang et al. 2013). The morphology of *B. corticulans* thallus, coupled with the optical properties of its filaments, creates a marked canopy effect on layers that are not directly hit by sunrays. The consequent filament self-shading and light screening cause a very effective functional segregation between algal filaments that switch into a prolonged dissipative state, and those underneath, which can maximise the capture of the attenuated light to ensure efficient photosynthesis. We can assume that such a separation gradually builds within the algal tufts in their natural habitat, and the extent and recovery of the observed sustained NPQ change. This explains why high *F*_v_/*F*_m_ values can be measured in the field after 30 min dark adaptation. In this context, it should also be noted that the in situ experiments took place during a window of hours in which the tidal level and light intensity varied over time. This ‘natural scenario’ clearly imposes a more variable excitation pressure on the algal filaments when compared with the controlled, extreme illumination provided in the laboratory (i.e. constant and prolonged, high illumination applied on filaments that were statically floating in a beaker). On one side, this is consistent with the average *F*_v_/*F*_m_ (~ 0.76) and its variability observed (range between ~ 0.69 and ~ 0.8) in the field, and on the other side with the lack of NPQ relaxation in the surface layers probed in the laboratory. Differently, when fluorescence changes are probed in the filaments 2 cm below the illuminated surface layer, almost no NPQ occurs. This means that, although the activation of sustained NPQ undermines photochemistry in the surface filaments, the inner layers of the algal thallus can maintain efficient photosynthetic rates. Furthermore, PSII yield in the surface filaments was ~ 0.4 during dark recovery (*F*_v_′′/*F*_m_′′), thus assuring ~ 40% efficiency of light energy use upon a subsequent submersion.

The presence of PsbS and LHCSR is debated in Bryopsidales (Christa et al. 2017; Handrich et al. 2017). No transcripts of PsbS and LHCSR have been found from transcriptomic analysis conducted on *Bryopsis hypnoides* and *C. taxifolia* (Handrich et al. 2017). Nonetheless, the expression of PsbS and LHCSR was reported and shown to be regulated by light changes in *Ulva linza* (Zhang et al. 2012) and *Ulva prolifera* (Mou et al. 2013). Although PsbS and LHCSR function is currently unknown in these species, these data indicate their possible involvement in the NPQ modulation in some Ulvophyceae. The presence of both proteins was therefore addressed in the present study, showing no light-induced transient monomerisation of PsbS and LHCSR3/LHCSR1 proteins in *B. corticulans*. Assuming a specific binding of the antibodies used, both types of proteins were detected as dimers or oligomers in *B. corticulans*, thus capable of withstanding solubilisation by SDS/LDS (see Bergantino et al. 2003). At the same time, primary antibody cross-reactivity to different protein sequences cannot be ruled out in *B. corticulans*. Hence, data interpretation must be careful.

The role played by monomeric and dimeric forms of PsbS and LHCSR proteins in NPQ is controversial. In plants, dimeric and monomeric PsbS has been found associated to LHCs in the thylakoid membrane, with dimers prevalently abundant at alkaline pH and monomers at acidic pH (Bergantino et al. 2003; Sacharz et al. 2017). Thus, it has been proposed that the PsbS dimer-to-monomer conversion is reversibly triggered by light and the presence of PsbS monomers is linked to NPQ formation (Bergantino et al. 2003; Sacharz et al. 2017). Nonetheless, PsbS homodimers have also been reported in the NPQ state of the antenna (Fan et al. 2015). Although LHCSR monomers have been usually reported during NPQ formation in *C. reinhardtii* and *P. patens* (e.g. Bonente et al. 2011; Pinnola et al. 2013), it has been recently speculated that finding LHCSR dimers in thylakoid membranes indicates the need for LHCSR3 activity of specific protein–protein interactions (Ballottari et al. 2016). However, no direct evidence for the photoprotective role of LHCSR dimers has been provided so far.

Given our results, as well as the sustained nature of NPQ observed, we propose that PsbS and LHCSR are not active in the NPQ mechanism of *B. corticulans*. Possibly, this is because they are either not functional (i.e. present as dimers or oligomers) or absent. The lack of the activity of allosteric regulators, such as XC and PsbS/LHCSR proteins, agrees with the absence of a fast and reversible qE in this species, as revealed here both in vivo and in isolated chloroplasts. This is consistent with a recent study on photoprotection of several Bryopsidales species (Christa et al. 2017). Although a ‘classical qE component’, characterised by fast development and reversibility, is missing in *B. corticulans*, we demonstrate that the transmembrane proton gradient is needed to initiate NPQ. Indeed, the absence of lumen acidification blocks NPQ induction in chloroplasts. Interestingly, our results find support from NPQ data collected on *B. hypnoides*, i.e. another intertidal macroalga that belongs to the same *genus* of the species here studied (Christa et al. 2017). A significant decrease in NPQ capacity is found when this alga is grown under low light and treated with nigericin (Christa et al. 2017). Differently, there is no obvious decrease in NPQ when it is cultured under high light, although the NPQ values measured are particularly low (~ 4 times less than those measured in low light-adapted algae). We believe that this might have hidden the potential impact of proton gradient disruption on NPQ formation in *B. hypnoides* (Christa et al. 2017).

ΔpH acts as a feedback signal linked to the degree of saturation of photosynthetic electron transport during light changes and controls induction and relaxation of the fast qE (Cardol et al. 2011; Ruban et al. 2012). Differently, in *B. corticulans*, lumen acidification seems to trigger only NPQ induction, which becomes persistent once activated. We hypothesise that antenna rearrangement (and possibly some uncoupling, suggested by the *q*_Pd_ rise) is involved in this sustained NPQ, which fits this species adaptation to the prolonged high light encountered during low tides. Measuring potential variations in PSII antenna cross section, and investigating structural and dynamic changes that underlie NPQ kinetics (e.g. freeze-fracture electron microscopy), could further unveil how the light-harvesting system of *B. corticulans* locks in into a long-term protective conformation.

Particularly outstanding is finding that the observed sustained NPQ, unrelated to violaxanthin de-epoxidation, is particularly effective in limiting the impairment/loss of PSII function, which is reflected as a decline in *q*_Pd_ below 0.98 (Ruban and Murchie 2012). Indeed, despite ~ 40% of RCs in the surface filaments is photodamaged by the prolonged high light illumination (*q*_Pd_ ~ 0.6), ~ 100% of RCs become open and functional after 40 min of darkness (*q* = 1.00, see Fig. 3e), whilst the high NPQ formed does not recover. This indicates and further supports the fact that NPQ components that slowly relax, and are often referred as qI, can still protect PSII RCs against photodamage (Ruban and Murchie 2012; Giovagnetti and Ruban 2015; Ware et al. 2015). Moreover, we show that high NPQ levels can be reached without the involvement of zeaxanthin, possibly relying on light-harvesting antenna rearrangement that allows conformational changes affecting the pigment–pigment and pigment–protein interactions responsible for quenching (Ruban 2016). These results suggest that either some transient photoinactivation of PSII, which does not require dismantling and repairing of photodamaged RCs, takes place, or the rate of PSII repair cycle is particularly fast in *B. corticulans*. Although at the moment we cannot exclude any of these hypotheses, further studies are required to elucidate *B. corticulans* resilience to photodamage.

In conclusion, we report that *B. corticulans* is able to cope with excess light energy by activating sustained protective NPQ. The siphonous cytology and morphology, and the peculiar molecular mechanics of NPQ adopted, enable this intertidal macroalga to efficiently protect itself, while still performing photosynthesis in the inner body filaments.

### *Author contribution statement*

VG, GH, MAW, TK, J-RS and AVR planned and designed the research; VG, GH, MAW, PU, XQ and WW performed the experiments. All authors analysed and interpreted the data. VG and AVR wrote the manuscript with contributions and inputs from all authors.
